# Characterization, expression profiles, intracellular distribution and association analysis of porcine *PNAS-4 *gene with production traits

**DOI:** 10.1186/1471-2156-9-40

**Published:** 2008-06-30

**Authors:** Delin Mo, Zhengmao Zhu, Marinus FW te Pas, Xinyun Li, Shulin Yang, Heng Wang, Huanling Wang, Kui Li

**Affiliations:** 1 State Key Laboratory of Animal Nutrition, Institute of Animal Science, Chinese Academy of Agricultural Science, Beijing 100094, PR China; 2 School of Life Science, Sun Yat-Sen University, Guangzhou 510006, PR China; 3 Department of Genetics and Cell biology, College of life sciences, Nankai University, Tianjin 300071, PR China; 4 Animal Breeding and Genomics Centre, Wageningen University and Research Centre, Lelystad, The Netherlands P.O. Box 65, 8200 AB Lelystad, The Netherlands

## Abstract

**Background:**

In a previous screen to identify differentially expressed genes associated with embryonic development, the porcine *PNAS-4 *gene had been found. Considering differentially expressed genes in early stages of muscle development are potential candidate genes to improve meat quality and production efficiency, we determined how porcine *PNAS-4 *gene regulates meat production. Therefore, this gene has been sequenced, expression analyzed and associated with meat production traits.

**Results:**

We cloned the full-length cDNA of porcine *PNAS-4 *gene encoding a protein of 194 amino acids which was expressed in the Golgi complex. This gene was mapped to chromosome 10, q11–16, in a region of conserved synteny with human chromosome 1 where the human homologous gene was localized. Real-time PCR revealed that *PNAS-4 *mRNA was widely expressed with highest expression levels in skeletal muscle followed by lymph, liver and other tissues, and showed a down-regulated expression pattern during prenatal development while a up-regulated expression pattern after weaning. Association analysis revealed that allele C of SNP A1813C was prevalent in Chinese indigenous breeds whereas A was dominant allele in Landrace and Large White, and the pigs with homozygous CC had a higher fat content than those of the pigs with other genotypes (*P *< 0.05).

**Conclusion:**

Porcine *PNAS-4 *protein tagged with green fluorescent protein accumulated in the Golgi complex, and its mRNA showed a widespread expression across many tissues and organs in pigs. It may be an important factor affecting the meat production efficiency, because its down-regulated expression pattern during early embryogenesis suggests involvement in increase of muscle fiber number. In addition, the SNP A1813C associated with fat traits might be a genetic marker for molecular-assisted selection in animal breeding.

## Background

Differentially expressed genes in the early stages of muscle development may be potential candidate genes to improve meat quality and quantity [1,2]. Recent studies showed that differential expression of genes during muscle development in prenatal pigs is associated with the differential stages of myogenesis [3,4], and expression patterns of these genes differ between pig breeds with different muscle characteristics [5,6].

An EST [GenBank: AA063650] was identified showing differential expression during the early stages of muscle development [7]. In order to identify this EST with expression associated with prenatal muscle development, a full-length cDNA library was constructed using 55-day Chinese Tongcheng pig fetus skeletal muscle [7]. From the cDNA library, a 2285-bp cDNA clone was obtained which was identical with this EST. After BLAST search in the non-redundant (nr) sequence database, it was found to be homologous to a clone DKFZp586C1019 [GenBank: AL049397] which is part of Homo sapiens *PNAS-4 *gene (also called *C1orf121*) encoding the CGI-146 protein. Recently, a report showed that human *PNAS-4 *is a novel pro-apoptotic protein activated during the early response to DNA damage [8]. Furthermore, this gene was also predicted as one of the targets of p53 tumor suppressor via mathematical modeling and quantitative data analysis [9]. Presently, Xenopus laevis *PNAS-4 *protein has been purified and confirmed by Western blot [10]. However, the biological functions of *PNAS-4*, especially the role in muscle development, are still unclear.

The aim of the present study was to characterize the porcine *PNAS-4 *gene by obtaining its sequence, and gain insight into its potential physiological role in muscle development related to meat production by studying subcellular localization, the temporal prenatal expression pattern, postnatal tissue expression levels and trait association analysis.

## Results

### Molecular cloning and sequence analysis

**Figure 1:**
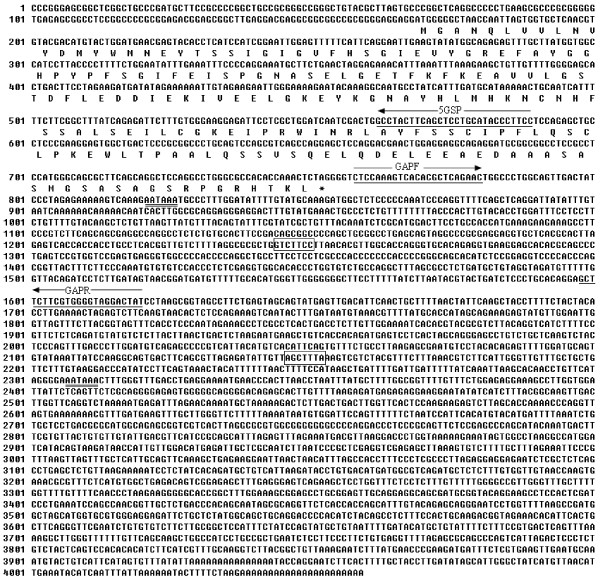
**Nucleotide and deduced amino acid sequence of porcine *PNAS-4***. The polyadenylation signals (AATAAA) are double-underlined; and the two elements named Brd-Box and GY-Box are boxed, separately. Primer site for 5' RACE assays is single underlined.

The full-length cDNA of porcine *PNAS-4 *gene consists of 4,059 bp that contains an open reading frame (ORF) of 582-bp encoding a protein of 194 amino acids with a calculated molecular mass of 21.4 kDa and an isolectric point (p*I*) of 4.807 (Figure 1). Two polyadenylation signals (AATAAA) were observed at positions 821 to 826 and 2307 to 2312 upstream of the poly (A) stretch. The Brd-Box (AGCTTTA) and GY-Box (GTCTTCC) were found in the 3' untranslated region (UTR) (Figure 1). The mRNA sequence of this gene has been submitted to GenBank [GenBank: DQ435075].

**Figure 2:**
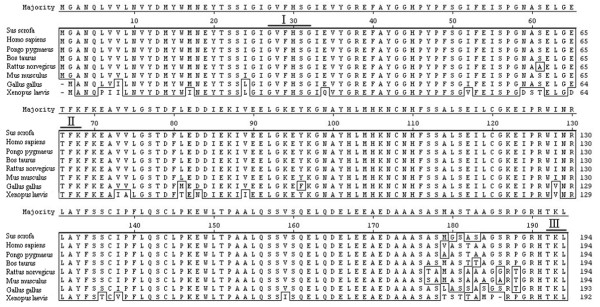
**Multiple amino acid sequence alignment of porcine CGI-146**. Different amino acid residues among species are presented with boxes. The three highly conserved motifs (N-myristoylation, Protein kinase C phosphorylation and MCTS) are indicated by roman letters (I – III), respectively.

Comparison of the porcine amino acid sequence with the proteins of seven species reported in GenBank shows that the porcine putative protein possesses a high level of identity (94–98%) with mammals and fowls, and it shares 74% identity with Xenopus laevis throughout the protein sequence. The porcine protein contained several conserved motifs such as C-terminal microbody targeting signal (CMTS), N-myristoylation and a Protein kinase C phosphorylation site (Figure 2). In addition, it showed that the porcine *PNAS-4 *encoded a non-secretory protein without signal peptide and transmembrane regions.

### Spatial and temporal expression pattern

**Figure 3:**
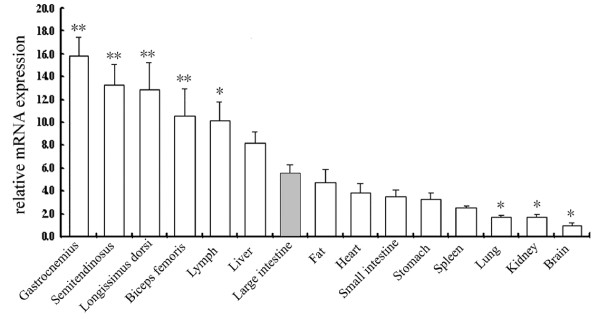
**The tissue distribution of porcine *PNAS-4 *mRNA assessed by qPCR**. The values shown in this figure are the averages of three independent experiments. Error bars represent the SD (*n *= 3) of relative mRNA expression levels of *PNAS-4 *normalized to *ACTB*. The values were normalized to endogenous *ACTB *expression and the value of *PNAS-4 *in brain was arbitrarily set to 1.0. In order to tell the detailed difference between the varied tissues, large intestine was used as a control. (* represents *P *< 0.05, ** represents *P *< 0.01).

The relative expression of the porcine *PNAS-4 *transcript in various pig tissues showed highest expression in skeletal muscle (in decreasing order: gastrocnemius, semitendinosus, longissimus dorsi and biceps femoris) and lymph node. Lower expression levels were determined in the liver, large intestine, backfat, heart, small intestine, stomach, spleen, lung, kidney and brain (Figure 3).

**Figure 4:**
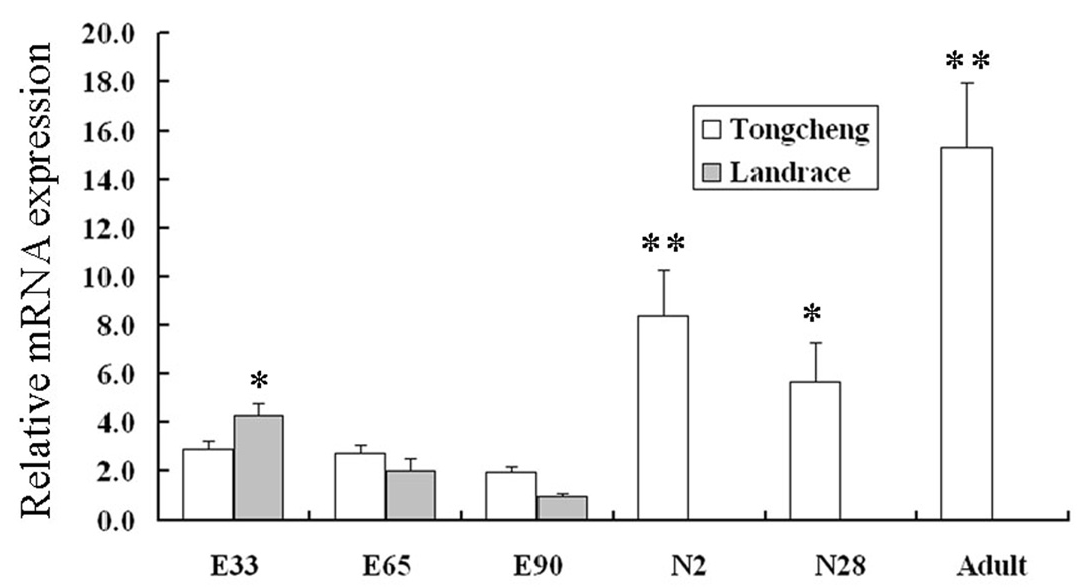
**The developmental profile of porcine *PNAS-4 *mRNA expression**. Porcine *PNAS-4 *mRNA expression levels in skeletal muscle normalized against *ACTB *during embryonic development and the postnatal period, measured by qPCR. Error bars represent SD (*n *= 3). Results were averaged from three independent assays during all stages observed. Symbols used are: *P < 0.05; **P < 0.01. E33: embryonic day 33; E65: embryonic day 65; E90: embryonic day 90; N2: muscle from 2-day neonate; N28: muscle from 28-day neonate.

Figure 4 showed that the expression level of porcine *PNAS-4 *seemed to decrease gradually during embryonic development of Landrace and Tongcheng pig breeds, which was significant in Landrace pigs between 33 days post conception (dpc) and 90 dpc. This was consistent with the results of semi-quantitative RT-PCR [7]. Curiously, after birth, the amount of porcine *PNAS-4 *mRNA in skeletal muscles increased drastically (*P *< 0.01). However, the mRNA expression on the day of weaning (28-day neonate) was lower than the second day after birth and adult (*P *< 0.05). After maturity, the mRNA expression of porcine *PNAS-4 *reached a peak level (*P *< 0.01).

### Intracellular distribution

**Figure 5:**
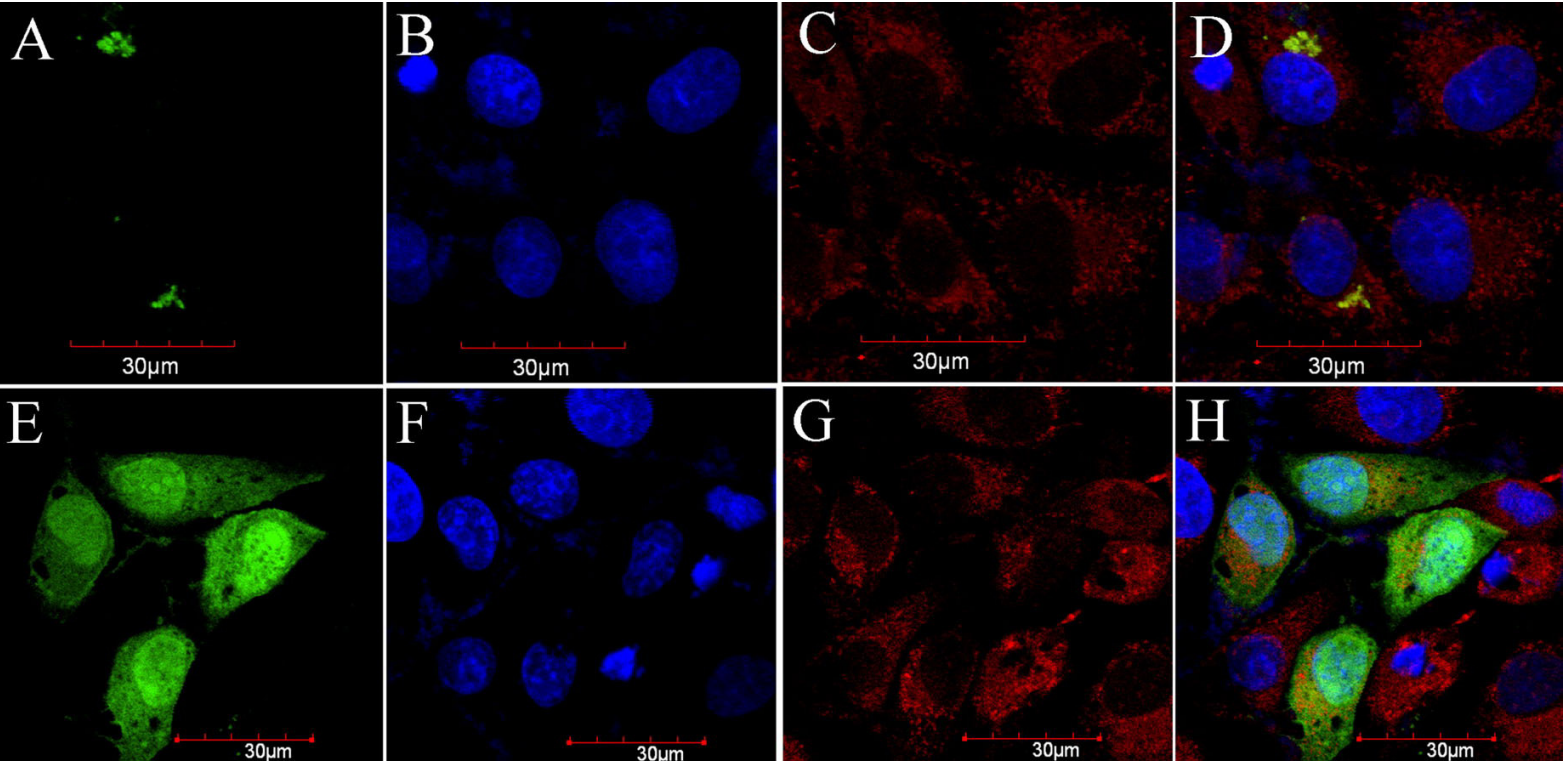
**Subcellular distribution of CGI-146 protein tagged with GFP**. The CGI-146-*GFP *fusion protein localized in Golgi complex marked with fluorescein isothiocyanate (A). Red fluorescence of mitochondria were stained with the mitochondrial-specific dye, Mito-Tracker Red (B) and blue fluorescence of nuclei were stained with Hoechst33342 (C). The overlay images were produced by merging all three signals together (D). The control micrographs (E-H) showed the GFP in the cytoplasm. Bars represent 30 *μ*M.

The CGI-146-GFP fusion proteins localized in the Golgi complex (Figure 5), which was identified by the complementation tests using the homo-functional P4-pEGFP-N3 construct. Green fluorescence was detected in the cytoplasm of the control cells transfected with pEGFP vector alone (E-H, Figure 5).

### Chromosome assignment

The somatic cell hybrid (SCH) panel revealed that the porcine *PNAS-4 *gene was assigned to SSC10 (q11–q16) (probability of regional localization: 0.9998, error risk < 0.1%). Two-point analysis of INRA-University of Minnesota porcine radiation hybrid (IMpRH) panel revealed that the porcine *PNAS-4 *was closely linked to SW497 (LOD = 13.68, 28 cRs) with 22% retention frequencies. This result was consistent with the SCH panel. The corresponding conserved syntenic region on human chromosome was the end of Homo sapiens chromosome 1 long arm (HSC1q)[11].

### Polymorphism detection and association with economic traits

Comparative sequencing revealed 9 and 5 polymorphisms within two PCR amplified fragments [GenBank: DQ406743 and DQ435075] which were parts of *PNAS-4 *genomic DNA. Because of linkage disequilibrium among several polymorphisms, two single nucleotide polymorphisms (SNPs) were selected for further study. The T1400C SNP within intron 2 was detectable after digestion with *Sty *I, resulting in a 762-bp PCR amplicon produced allele T (762 bp) and allele C (549 bp and 213 bp). The other SNP, A1813C within the 3'UTR, located more than 20 kb away from the T1400C SNP in genome, was harbored in a 789-bp PCR amplicon and digested by *Msp *I, resulting in allele A (789 bp) and allele C (606 bp and 183 bp).

Analysis of polymorphism revealed that allele C for both polymorphisms (T1400C and A1813C) was prevalent in four Chinese indigenous pig breeds whereas the allele T (T1400C) and the allele A (A1813C) respectively had higher frequencies in Landrace and Large White (Table 1). The two SNPs could be linked in Landrace, Large White pigs and a pair of three breed-crosses (data not shown). Therefore, we selected one of the two SNPs for association analysis. Three significant associations were observed between the A1813C SNP genotypes and three fat-related traits (Table 2). For the three considered traits, homozygous CC pigs of our experimental population showed higher values than those of the pigs with other genotypes (*P *< 0.05).

**Table 1:** Genotypes of porcine SNPs T1400C and A1813C in different pig breeds

Pig Breeds	No.	T1400C (*Sty *I)			A1813C (*Msp *I)		
		
		*TT*	*TC*	*CC*	*AA*	*AC*	*CC*
Wuzhishan	43	0	0	43	7	18	18
Xiang	42	0	21	21	8	13	21
Bamaxiang	45	0	14	31	0	0	45
Tongcheng	44	1	11	32	5	11	28
Landrace	15	12	0	3	12	0	3
Large White ^a^	19	15	0	4	15	0	4
Large White ^b^	196	139	54	3	139	54	3

^a ^DNA samples collected from our experimental pig group.

^b ^DNA samples collected from two experimental Large White selection lines originating from one commercial population.

**Table 2:** Association analyses between SNP A1813C and three fat-related traits

	SNP A1813C Genotype (mean ± SE)			P-value		
	
Traits	AA (67)	AC (52)	CC (37)	AA-AC	AA-CC	AC-CC
Percentage of leaf fat	2.794 ± 1.31	3.259 ± 1.2	5.261 ± 1.44	0.847	0.008**	0.017*
Percentage of leaf and caul fat	5.010 ± 2.29	5.928 ± 2.6	9.933 ± 2.96	0.806	0.012*	0.028*
BFT between 6^th ^and 7^th ^ribs	3.257 ± 0.93	3.199 ± 0.71	4.262 ± 0.93	0.183	0.324	0.039*

* represents *P *< 0.05, ** represents *P *< 0.01

## Discussion

Differential expression of genes during a biological process suggests a direct or indirect relationship between the genes and the biological process[12]. An EST [GenBank: AA063650] was identified showing differential expression during the early stages of muscle development [7]. Therefore, the full-length cDNA of the *PNAS-4 *gene was obtained and analyzed for study its structure and biological functionality. This manuscript reports the cDNA and amino acid sequences of the gene, its mRNA expression profiles, protein localization, and functional analysis in muscle tissue formation and meat production.

### *PNAS-4 *cDNA and amino acid analysis

Full-length cDNA sequence analysis revealed high identity of the gene at both the mRNA and the amino acid level with mammalian and avian species. This may indicate both structural and functional conservation of the gene. The Brd-Box and GY-Box within the 3'UTR suggests that the *PNAS-4 *expression was regulated negatively at the post-transcriptional level, both temporal and spatial [13-15].

### Functional analysis in muscle tissue related to meat production

*PNAS-4 *mRNA showed a widespread expression with the highest levels in muscle tissue, and could be detected at all times during the skeletal muscle development. In more detail, the expression of *PNAS-4 *tends to decrease gradually from prenatal day 33 to day 90, an important period for development of muscle fibers and determination of muscle fiber numbers [1,16,17]. The muscle fiber number appears to increase as a result of the decrease of *PNAS-4 *mRNA expression level during prenatal muscle development, which was consistent with that the *PNAS-4 *is involved in apoptosis[8]. However, the expression profiles of Tongcheng pigs and Landrace pigs were not complete similar in early embryonic development, and this kind of difference may be related to breed difference [5]. The *PNAS-4 *mRNA expression decreased faster in early Landrace pig development, and this result may be correlated with the high number of primary fibers found in Landrace compared with Tongcheng pigs and other indigenous Chinese breeds [18-21]. In other words, there might be a positive correlation between the decrease speed of *PNAS-4 *mRNA expression level in embryonic stage and porcine muscle fiber number. Muscle fiber number is an important determinant of postnatal growth such that pigs with a high fiber number tend to grow faster than pigs with a lower fiber number [22]. This is also supported by the carcass weight of Landrace compared to Tongcheng pigs[18]. After birth, the expression of *PNAS-4 *mRNA in muscle tissue was increased, which could be attributed to an increase in length and girth of the muscle fibres and/or muscle function [1]. Consistent with this, the highest expression of the gene was recorded in skeletal muscle of mature pigs. The protein was found to localize in the Golgi complex. It was thus deduced that *PNAS-4 *protein acted more possibly as a regulator than as a structural protein, which was generally in agreement with the opinion that *PNAS-4 *may be a pro-apoptotic gene [8].

### *PNAS-4 *as a candidate gene or QTL for fat deposition

Porcine *PNAS*-4 gene mapped to q11–16 of SSC10, closely linked to marker SW497. It showed that several quantitative trait loci (QTL) related to growth and carcass traits, such as average daily gain (ADG), Backfat Thickness (BFT), Carcass Weight (CCWT), Ham Weight (HAMWT), are located on this chromosome region in the Pig QTL Database release [23-25]. Consistent with this, significant associations between A1813C SNP genotypes of *PNAS-4 *and three fat-related traits were found in our experimental pig populations. Moreover, the three fat traits values of pigs with homozygous CC were higher than that of the pigs with other genotypes (*P *< 0.05). Our findings have also revealed that allele C of SNP A1813C was prevalent in four Chinese indigenous breeds whereas allele A had higher frequency in Landrace and Large White. Interestingly, western commercial pig breeds (Duroc, Large White and Landrace) are well known for their high growth rate and lean meat percentage [26]. These correlated results suggest that the pigs with allele C in SNP A1813C will have higher fat content. In order to confirm it, further investigations in the use of the gene for commercial breeding are recommended.

## Conclusion

We cloned and characterized the full-length cDNA of porcine *PNAS-4 *gene. Expression profiles and gene structure suggest that this gene was regulated negatively at the post-transcriptional level, both temporal and spatial. It might play a negative role in modulating muscle fiber numbers, and act as an important factor affecting the muscle mass efficiency. Unexpectedly, association analysis revealed that allele C of SNP A1813C was prevalent in four Chinese indigenous breeds whereas allele A had higher frequency in Landrace and Large White, and the pigs with allele C in SNP A1813C will have higher fat content. It is a potential genetic marker for animal breeding.

## Methods

### cDNA isolation and sequence analysis

BLAST in the pig expressed sequence tags (ESTs) database using the human *PNAS-4 *mRNA sequence [GenBank: NM 016076] as probe, revealed three porcine ESTs [GenBank: CN156918, CV877591 and AJ665351]. To obtain the full-length cDNA, primer set GAPF/GAPR (Table 3) was used to amplify the unknown nucleotide sequence between ESTs CV877591 and AJ665351, and the primer 5GSP (Table 3) in combination with the Universal Primer A Mix (UPM) supplied in the SMART™ RACE Kit (Clontech Inc, Palo Alto, CA, USA) used to amplify the 5'-end of the gene. Rapid amplification of cDNA ends (RACE) PCR was carried out in a PTC-200 thermal cycler (Bio-Rad, Richmond, CA, USA) with touchdown PCR conditions: 3 min at 95°C; 5 cycles of 94°C for 20 s, 72°C for 3 min; 5 cycles of 94°C for 20 s, 70°C for 30 s; 72°C for 3 min; 32 cycles of 94°C for 20 s, 68°C for 30 s; 72°C for 3 min; 5 min at 72°C. The PCR products were purified by agarose gel electrophoresis and sequenced. Functional elements analysis of the putative protein and UTR were performed using the PROSITE program[27], SignalP 3.0 server[28] and UTRScan[29], respectively.

**Table 3:** Primers and probe employed in these experiments

Function	Primer name	Primer and probe sequences (5'-3')	Tm
Cloning	GAPF	CTCCAAAGTCACACGCTCAGAAC	62
	GAPR	ATAGTCCTACCCCACGAAGAAGC	
	5GSP	GGAAGGGTATGCAGGAGCTGAAGTAGGC	71
Subcellular	P4GFPF	CGGCTCGAGGGATGGGGGCTAACC	63
localization	P4GFPR	GAGAAGCTTAGAGTTTGGTGTGGCG	
Real-time	Real-time F	CCCAGGAAATGCTTCTGAACTAG	60
PCR	Real-time R	CCCACAAAGAATCTCTGATAAAGC	
	ProbeP4	AAGCTGTTGTTTTGGGGAGCACTG	
	*ACTB *F	GGATGCAGAAGGAGATCACG	60
	*ACTB *R	CTCGTCGTACTCCTGCTTGC	
	ProbeBeta	ATCAAGATCATCGCGCCTCCCGAGC	
Mapping	MapF	TGGCAGAGCGGTCGTCACTTAGGC	65
	MapR	GAGCAGAATCTCCTCCCACGCACCAT	
SNPs detection	SNPF1	CTAGAACCACTCAAACCAAGCAGC	62
	SNPR1	ATCAGGCAGGTAAAAGGATAACGG	
	SNPF2	GCCTTCTGAGTAGCAGTATGAGTTG	62
	SNPR2	CCTGCGAGAACTGAGAATAATCC	

### QPCR analysis of gene expression patterns

Fifteen tissues (stomach, large intestine, kidney, lymph node of neck, small intestine, liver, heart, longissimus dorsi muscle, lung, gastrocnemius, biceps femoris muscle, brain, backfat, semitendinosus and spleen) were obtained from three Chinese indigenous Wuzhishan (a Chinese mini pig breed) sows. Three pig embryos were collected from every pregnant female during three embryonic periods (33, 65, and 90 dpc) and three postnatal periods (2, 28 days and adult (160 days)). The longissimus dorsi muscle were harvested, mixed, frozen in liquid nitrogen, and stored at -80°C. Furthermore, three Landrace pigs were employed to determine expression differences between western and Chinese pig breeds in prenatal stages. All animal procedures were performed according to guidelines developed by the China Council on Animal Care and protocol approved by Animal Care and Use Committee of Hubei Province, PR China.

QPCR was performed with the Chromo4™ Real-Time PCR Detector (Bio-Rad) using gene-specific primers and Taqman probes (Table 3). Total RNA (4 *μ*g), extracted from tissues using the Trizol reagent (Invitrogen, Carlsbad, CA, USA) and treated with RNase-free DNase (MBI Fermentas, St. Leon-Rot, Germany). RNA quality was assessed with agarose gel electrophoresis and the ND-1000 Spectrophotometer (NanoDrop Technologies, USA), and was discarded if the 260/280 ratio was not between 1.8 and 2.1. It was reverse-transcribed into cDNA using M-MLV reverse transcriptase (Promega Corp., Madison, WI, USA). cDNA synthesis was performed in duplicate. Both beta-actin gene (*ACTB*) and glyceraldehyde-3-phosphate dehydrogenase (*GAPDH*) were consistently expressed in all tissues of this assay, so we selected the *ACTB*, one of the two housekeeping genes, as an internal control for normalization purposes. The minus RT control with primers for *ACTB *was performed to test if products were derived from DNA. Relative transcript quantification was performed using standard curves generated for *ACTB *and *PNAS-4 *gene from a 10-fold serial dilution of cDNA. Pooled cDNA from a subset of the gastrocnemius samples examined in this study was used to generate the standard curves. In this assay, the efficiency of *ACTB *and *PNAS-4 *gene primers were 96.5% and 96.95%, respectively. The cycling conditions consisted of an initial, single cycle of 95°C for 3 min, followed by 35 cycles of 15 s at 94°C and 1 min at 60°C. Each reaction (in 20 *μ*l) contained 1 × PCR buffer (TaKaRa, Dalian, P. R. C.), 0.4 *μ*M gene-specific primers and 0.2 *μ*M probe, 50 *μ*M of each dNTP, 3 mM MgCl_2_, 2.0 U Taq DNA polymerase (TaKaRa) plus 2 *μ*l normalized template cDNA. All PCR amplifications were performed in triplicate for each RNA sample and gene expression levels were quantified relative to *ACTB *expression using Gene Expression Macro software (Bio-Rad). The results were analyzed using the 2^-ΔΔCt ^method described previously[30]. Differences in gene expression between groups were evaluated using Student's t-test and were considered statistically significant at *P *< 0.05.

### Intracellular distribution of the porcine *PNAS-4 *gene

The intracellular distribution of porcine *PNAS-4 *protein was studied by fluorescence and confocal analysis of pig kidney epithelial cells (PK15) cells transiently transfected with P4-pEGFP-C1 construct. The ORF, encoding porcine *PNAS-4 *protein, was amplified by PCR using the primers (P4GFPF/P4GFPR, Table 3) designed from its cDNA sequence and subcloned into the *Xho *I-*Hind *III site of the pEGFP-C1 vector (BD Biosciences Clontech, CA, USA).

PK15 cells were cultured in Dulbecco's modified Eagle's medium (DMEM) supplemented with 10% fetal calf serum (GIBCO-BRL, Grand Island, NY), 4 mM glutamine, 100 U/ml penicillin, and 0.1 mg/ml streptomycin under humidified air containing 5% CO_2 _at 37°C and seeded onto cover slips in 6-well plates. The PK15 cells were plated at 5 × 10^5^/ml and transfected with P4-pEGFP-C1/N3 constructs (1 *μ*g of plasmid DNA) using Lipofectamine™ 2000 reagent according to the manufacturer's instructions until they were grown to 80–90% confluence.

Twenty-four hours after transfection, the cells were incubated at 37°C for 30 min in growth medium containing 200 nM MitoTracker Red CM-H_2_Xros (Molecular Probes, Eugene, Oregon, USA) for mitochondrial labeling. Then the cells were fixed with pre-warmed growth medium containing 3.7% formaldehyde for 15 min at 37°C. After the final washing steps and incubation with 10 *μ*M Hoechst33342 for 10 min, the slides were mounted, sealed and analyzed by Olympus FluoView™ FV1000 Confocal Microscope. The software FV1000 Viewer was used to generate individual fluorescent pictures as well as overlay pictures that demonstrated the relative distribution of the fusion protein.

### Chromosome mapping by SCH and IMpRH

The French SCH panel composed of 27 pig/rodent hybrids [31] was used for the regional assignment of *PNAS-4 *gene on the SSC. To more precisely map genes, the IMpRH panel consisting of 118 hybrid clones obtained by fusing irradiated pig cells with the recipient hamster cell was employed [32]. The PCR was performed in a total volume of 10 *μ*L 1 × PCR buffer (TaKaRa), containing 25 ng panel DNA, 0.3 *μ*M each primer (MapF, MapR, Table 3), 75 *μ*M each dNTP, 1.5 mM MgCl_2_, and 1.0 U Taq DNA polymerase (TaKaRa). The PCR cycling conditions were: Initial denaturation at 95°C for 3 min, followed by 32 cycles (20 s at 94°C, 30 s at 65°C and 30 s at 72°C). Statistical analysis of SCH PCR results were performed using the program [33]available for regional assignment of genetic markers. The results of radiation hybrid PCR products were analysed with the IMpRH mapping tool [34].

### Polymorphism detection and association with meat traits

Four Chinese indigenous pig breeds (Xiang (N = 42), Bamaxiang(N = 45), Wuzhishan (N = 43) and Tongcheng (N = 44) pigs) and two western white breeds (Large White (N = 215) and Landrace (N = 15)) were used to analyze genetic variability. Association analyses were performed in our experimental populations that contained three pure breeds, Tongcheng (N = 44), Landrace (N = 15) and Large White^a ^(N = 19), and a pair of three-breed cross, Landrace × (Large White × Tongcheng) (N = 44) and Large White × (Landrace × Tongcheng) (N = 35). Growth traits (average daily gain), carcass and meat quality traits (live weight at slaughter, carcass length, dressing percent, loin muscle area, meat color, marbling, drip loss, 0 h loin muscle PH and shear force) were studied. Fat traits studied were percentage of leaf fat, percentage of leaf and caul fat, and backfat thickness between 6th and 7th ribs.

Two pairs of primers (SNPF1/SNPR1 located in intron 2 and SNPF2/SNPR2 located in 3'UTR) were designed using the submitted sequences [GenBank: DQ406743 and DQ435075]. The PCR products were cloned into the T-easy vector and sequenced to identify the polymorphisms in the porcine *PNAS-4 *gene.

Associations between growth traits, carcass traits and meat quality traits on the one hand and genotype on the other hand were studied using a two-step analysis method. Briefly, in the first step, a preliminary general linear model without the genotype information was used to eliminate system effects, including sex, combination and batch, using the SPSS program with the following model:

*Y*_*ijkl *_= *μ *+ *C*_*i *_+ *B*_*j *_+ *S*_*k *_+ (*CB*)_*ij *_+(*BS*)_*jk *_+ *e*_*ijkl*_

where *Y*_*ijkl *_is phenotypic value of the target trait, *μ *is the population mean, *C*_*i *_is the combination (including breed) effect, *B*_*j *_is the batch effect, *S*_*k *_is the sex effect and *e*_*ijkl *_is random error effect for each observation. In addition, the interaction effects between combination and batch (*CB*)_*ij*_, and between batch and sex (*BS*)_*jk*_, were also considered. In the second step, the resulting standardized residual values were used to partition the effects of the genotypes using the one-way analysis of variance (ANOVA) model. Simultaneously, significant difference tests between different genotypes were performed with Bonferroni *t *tests [35].

## Abbreviations

dpc: days post conception, ESTs: expressed sequence tags, GFP: green fluorescent protein, HSC: homo sapiens chromosome, IMpRH: INRA University of Minnesota porcine radiation hybrid, ORF: open reading frame, PK15: Pig Kidney Epithelial cells, QTL: quantitative trait loci, RACE: rapid amplification of cDNA ends, SCH: somatic cell hybrid, SNPs: single nucleotide polymorphisms, SSC: sus scrofa chromosome, UTR:  untranslated region.

## Authors' contributions

DM performed research and drafted the manuscript. ZM participated in the design and the realization of the experiment. MFWtP provided DNA samples for SNP genotyping, and finalized the writing. XL participated in cell culture and subcellular localization. SY was responsible for the data management. HW contributed to bioinformatics analysis. HLW participated in SNP identification. KL conceived and coordinated the study and corrected the manuscript.
